# Exploring the complex interplay: gut microbiome, stress, and leptospirosis

**DOI:** 10.3389/fmicb.2024.1345684

**Published:** 2024-02-27

**Authors:** Pavlo Petakh, Valentyn Oksenych, Iryna Kamyshna, Iryna Boisak, Katerina Lyubomirskaya, Oleksandr Kamyshnyi

**Affiliations:** ^1^Department of Biochemistry and Pharmacology, Uzhhorod National University, Uzhhorod, Ukraine; ^2^Department of Microbiology, Virology, and Immunology, I. Horbachevsky Ternopil National Medical University, Ternopil, Ukraine; ^3^Broegelmann Research Laboratory, Department of Clinical Science, University of Bergen, Bergen, Norway; ^4^Department of Medical Rehabilitation, I. Horbachevsky Ternopil National Medical University, Ternopil, Ukraine; ^5^Department of Childhood Diseases, Uzhhorod National University, Uzhhorod, Ukraine; ^6^Department of Obstetrics and Gynecology, Zaporizhzhia State Medical and Pharmaceuticals University, Zaporizhzhia, Ukraine

**Keywords:** leptospirosis, gut microbiome, stress, post-traumatic stress disorder, military personnel, war zones, T-lymphocytes

## Abstract

Leptospirosis, a re-emerging zoonotic disease, remains a significant global health concern, especially amid floods and disasters such as the Kakhovka Dam destruction. As is known, the stress that occurs in the conditions of military conflicts among civilian and military personnel significantly affects susceptibility to infectious diseases and possibly even influences their course. This review aims to explore how the gut microbiome and stress mediators (such as catecholamines and corticosteroids) might impact the leptospirosis disease course. The review opens new horizons for research by elucidating the connections between the gut microbiome, stress, and leptospirosis.

## 1 Introduction

Leptospirosis, caused by the pathogenic spirochetes of the Leptospira genus, remains a significant global health concern and is classified as a re-emerging zoonotic disease (Ko et al., 2009; Petakh et al., 2022a; Bradley and Lockaby, 2023). With an estimated annual infection rate of more than a million individuals and a staggering 60,000 associated deaths, leptospirosis imposes a substantial burden on public health worldwide (Haake and Levett, 2015; Samrot et al., 2021; Petakh and Nykyforuk, 2022; Petakh et al., 2022b, 2023a). This infectious disease primarily spreads through contact with the urine of infected hosts, often found in contaminated water sources or within the soil. Leptospirosis can manifest in a spectrum of clinical presentations, ranging from mild or asymptomatic cases to severe, life-threatening conditions that involve multi-organ dysfunction (Petakh et al., 2022c). Weil’s syndrome is a critical and potentially fatal manifestation of the disease that is characterized by renal, pulmonary, and hepatic impairment (Latchoumi et al., 2020; Abdullah et al., 2021). Notably, Weil’s syndrome carries a high mortality rate and is characterized by hepatic dysfunction, renal failure, and hemorrhagic complications (Limothai et al., 2021). In the face of such a formidable disease, the early recognition and provision of intensive medical care are paramount to improving patient outcomes.

While leptospirosis already presents a formidable challenge, certain populations are particularly susceptible to its risks. The ongoing conflict in Ukraine has placed military personnel in a unique position, where they face heightened exposure to the elements that can facilitate leptospiral transmission (Petakh & Kamyshnyi, 2023; Petakh et al., 2023b). Frequent contact with water sources and potential reservoir hosts, such as rodents, elevates the risk of infection among these individuals (Brinker and Blazes, 2017). Furthermore, military personnel operate under conditions that are often associated with elevated stress levels (Bray et al., 2001).

The civilian population is also potentially in danger of leptospirosis. The destruction of the Kakhovka Dam on 6 June 2023, has caused widespread devastation and human suffering. In the short term, there is a significant risk of rodent-borne diseases such as leptospirosis and tularemia (Wilkenfeld and Berenji, 2024). In the medium to long term, the World Health Organization (WHO) is concerned about the lasting physical and mental health impacts on affected communities, the environmental harm caused by the floods, and damage to health facilities, which may reduce access to essential and specialized services (WHO, 2023). Confirmation of the risks of local outbreaks of leptospirosis is evident in the increased incidence of leptospirosis in 2023. Center for Public Health of the Ministry of Health of Ukraine reported a 3.1-fold increase in the number of leptospirosis patients in 2023 compared to the previous year (WHO, 2023). The study by Oleh Lushchak et al. involving 3,173 Ukrainians found that moderate and high stress was prevalent among 68.2% and 15.5% of NDPs, 64.4% and 21.6% of IDPs, and 64.7% and 25.2% of refugees, respectively (Lushchak et al., 2024). Stress can also dysregulate humoral and cellular immune responses to pathogens, increasing risk for infectious illnesses including influenza and the common cold (Glaser and Kiecolt-Glaser, 2005; Seiler et al., 2020). The association between psychological stress and susceptibility to the common cold has long been recognized; stress suppresses the host resistance to infection and increases rates of infection (Cohen et al., 1991).

As a result, a compelling need exists to comprehensively explore the relationship between stress and leptospirosis, with a particular focus on the intricate interplay involving gut microbiota and T-lymphocytes. This review aims to elucidate the complex dynamics of stress and its potential impact on leptospirosis, shedding light on a multifaceted connection that bears implications for the health and well-being of military personnel and beyond.

Understanding the relationship between stress and leptospirosis is crucial due to the high prevalence of moderate and high stress among NDPs, IDPs, refugees, and military personnel. By examining the interplay between gut microbiota and T-lymphocytes, this review aims to provide insights into the complex dynamics of stress and its potential impact on leptospirosis. This multifaceted connection has implications not only for military personnel but also for the overall health and well-being of the civilian population.

## 2 Leptospira immunity and pathogenesis of leptospirosis

Leptospira infection initiates with the attachment of bacteria to host cells and the formation of pores. Virulence factors, either surface-present or secreted by the bacteria, play a crucial role in this process (Barbosa et al., 2006; Cinco et al., 2006). These factors aid in attachment and may be involved in forming pores or causing host cell lysis. Leptospira surface proteins, like LigB and LipL32, help the organism attach to different host cells and parts of the extracellular matrix (Banfi et al., 1982; Choy et al., 2007).

Upon infection, the innate immune system is activated by recognizing Microbial Pathogen-Associated Molecular Patterns (PAMPs) through Pattern Recognition Receptors (PRRs) like Toll-like receptors (TLRs) and nucleotide-binding oligomerization domain (NOD)-like receptors (NLRs; Akira et al., 2006; Mogensen, 2009). This recognition triggers inflammatory responses mediated by signaling pathways like NF-κB and activator protein 1 (AP-1). Pro-inflammatory molecules like cytokines, prostaglandins (PGs), and Nitric Oxide (NO) are produced, leading to increased arterial dilation and vascular permeability (Petrilli et al., 2005; Turner et al., 2014; Cagliero et al., 2018).

Interestingly, leptospiral lipopolysaccharide (LPS) activates TLR2 in human cells, unlike the classical TLR4 activation seen with other bacterial LPS. In mice, both TLR2 and TLR4 are activated by leptospiral LPS (Nahori et al., 2005). An essential defense mechanism during the initial infection is the activation of the alternative complement pathway, providing resistance against the complement system, especially in virulent strains. Recent research suggests that leptospires can avoid recognition by specific Toll-like and NOD-like receptors, possibly influencing susceptibility to leptospirosis in different hosts (Bonhomme and Werts, 2022).

Leptospires, being extracellular pathogens, elicit an acquired immune response involving antibody production and activation of the classical complement pathway (Fraga et al., 2011). Opsonization by specific IgG antibodies enhances phagocytosis by neutrophils and macrophages (Wang et al., 1984). Leptospires, however, can evade the immune response by switching complement pathways through their proteins, like adhesins and endostatins (Banfi et al., 1982). They can colonize various tissues, with a preference for the kidney due to the absence of a complement pathway (Abdullah et al., 2021).

While the host activates T-cell responses against the infection, the effectiveness seems insufficient to prevent the infection. Leptospires can invade various tissues and move between them but tend to colonize the kidney, utilizing immune evasion strategies.

## 3 Microbiota under stress: unveiling the gut-brain axis

**Figure 1:**
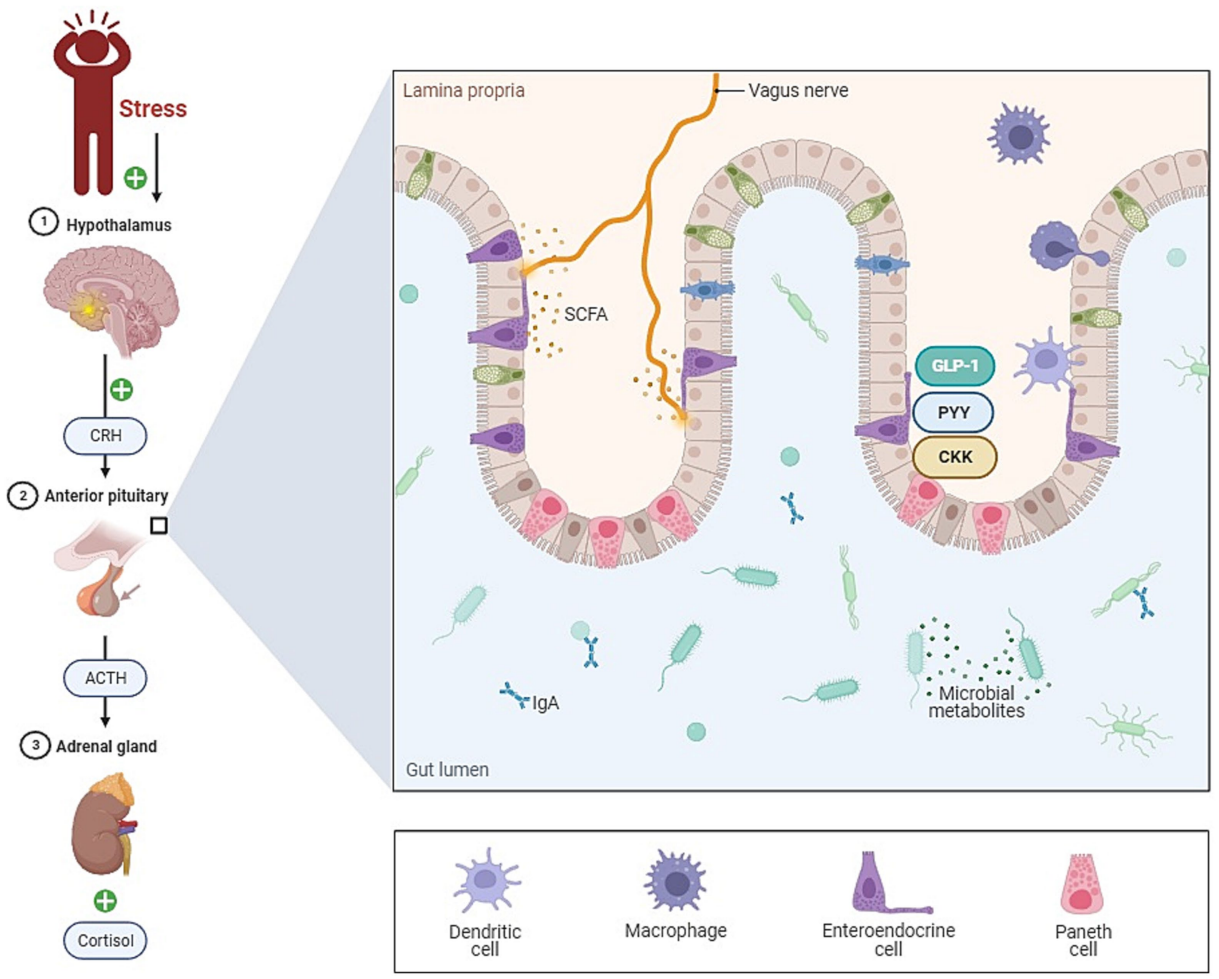
The role of gut microbiota in stress (gut-brain axis). This figure illustrates the complex relationship between the gut and the brain, known as the gut-brain axis. Key components include the vagus nerve, short-chain fatty acids (SCFA), and immune cells within the lamina propria of the gut. The vagus nerve serves as a vital communication link between the gut and the brain, facilitating bidirectional signaling. Changes in gut microbiota composition can profoundly impact immune cells in the lamina propria, leading to alterations in immune responses and potentially triggering inflammation. This dysregulation in the gut immune system is a critical aspect of the pathogenesis, highlighting the role of the gut microbiota in influencing stress-related responses.

The gut microbiome has emerged as a significant factor that may influence stress resilience (Figure 1). Over the past decade, we have come to appreciate the profound impact of the gut microbiota on human health, including its role in psychiatric well-being (Cryan and Dinan, 2012). The gut-brain axis represents a bidirectional channel of communication between the gut and the central nervous system, playing a pivotal role in maintaining neural, hormonal, and immunological equilibrium (Carabotti et al., 2015). With mounting evidence indicating that the gut microbiome can affect symptoms of depression and anxiety, it is now recognized as a crucial element in the cross-talk between the gut and the brain, leading to the extended concept of the microbiome-gut-brain axis (MGBA; Bear et al., 2021).

Correlational studies have demonstrated distinct differences in fecal microbiota composition between individuals with anxiety or depression, including those in remission, and their healthy counterparts (Naseribafrouei et al., 2014; Jiang et al., 2015; Navarro-Tapia et al., 2021). Women with a higher abundance of *Prevotella* in their feces have shown increased negative emotional responses to negative stimuli and lower brain activity in the hippocampus compared to those with a higher abundance of *Bacteroides* (Tillisch et al., 2017). Several rodent studies have experimentally established that the presence and composition of the gut microbiota can influence emotional behavior. In mice, gut infections or inflammation have been associated with patterns of behavior indicative of anxiety, such as reduced exploration and increased behavioral inhibition (Lyte et al., 1998, 2006; Bercik et al., 2010). Germ-free (GF) rats and mice, born and raised without microbiota, exhibit either increased or decreased anxiety and depressive-like behaviors when compared to counterparts with specific pathogen-free (SPF) gut microbiota (Clarke et al., 2013; Nishino et al., 2013; Crumeyrolle-Arias et al., 2014). Some probiotics have also demonstrated mood-altering effects, with the term “psychobiotics” referring to probiotics that confer mental health benefits through interactions with commensal gut bacteria (Sarkar et al., 2016).

However, MGBA research is not without inconsistencies and challenges. Findings from MGBA studies do not consistently align, and the translation of results from animal studies to human research has been a concern. Animal behavioral tests have inherent limitations in mirroring anxiety- or depressive-like symptoms in humans, and human studies encounter methodological challenges due to the heterogeneity of lifestyles and difficulties in collecting specific biological samples such as colon microbiota and host tissues.

Probiotic supplementation has yielded mixed effects on emotional behavior. Some rodent studies have shown a reduction in anxiety-like or depressive-like behaviors following probiotic supplementation, especially in cases of inflammation-induced behavioral changes and stress-induced behavior alterations (Desbonnet et al., 2010; Bravo et al., 2011; Smith et al., 2014; D'Mello et al., 2015; Liang et al., 2015; Cryan et al., 2019). However, other studies have found no significant differences, and translational research has produced variable results as well. In some cases, probiotic supplements did not influence mood in healthy adults or individuals with irritable bowel syndrome, while other studies reported reduced depression scores in individuals with diagnosed depression and healthy adults, or decreased emotional reactivity (Desbonnet et al., 2008; Simrén et al., 2010; Messaoudi et al., 2011; Steenbergen et al., 2015; Akkasheh et al., 2016; Barrera-Bugueño et al., 2017; Kelly et al., 2017). A mixed outcome was observed in a study where anxiety scores were reduced but not depression scores, and mood improvement was evident only in those with low baseline mood (Benton et al., 2007; Rao et al., 2009).

Moreover, the direction of change in anxiety-like behaviors in GF rodents appears to depend on the strain, with stress-sensitive strains exhibiting increased anxiety-like behaviors and more resilient strains showing decreased anxiety-like behaviors. The presence and composition of the gut microbiota impact mood, with potential variations in host genotype and phenotype playing a crucial role in determining whether changes in the gut microbiota affect mood. The mechanisms underlying MGBA are intricate, intertwined, and bidirectional, and various factors, such as diet, stress, exercise, and individual experiences, may result in different mechanisms of mood modulation.

An emerging area of interest revolves around the interactions between stress and the MGBA. Stress can induce alterations in the gut microbiota composition, as demonstrated in rodent models of psychological stress (Bailey et al., 2011; Galley et al., 2014a,b, 2017; Burokas et al., 2017; Marin et al., 2017; Yang et al., 2017; Gautam et al., 2018; Tsilimigras et al., 2018). Stress during pregnancy has also been shown to reshape the gut microbiome structure in offspring as well as in dams (Jašarević et al., 2017). Given the growing evidence that changes in the gut microbiota can influence mood, it is plausible that stress-induced alterations in the gut microbiota might contribute to the development of chronic stress, anxiety, and depression following a stressful event. Conversely, mitigating stress-induced changes in the gut microbiota and their physiological effects could potentially enhance stress resilience.

Changes in the gut microbiota following stress exhibit considerable variation among studies, with most evidence derived from rodent research. These changes include a reduction in the relative abundance of *Lactobacillus* and an increase in genera containing opportunistic pathogens like *Odoribacter*, *Clostridium*, and *Mucisprillum*, as well as a decrease in *Bifidobacterium* although one study reported an increase in stress-resilient mice (Holdeman et al., 1976; Galley et al., 2014b; Marin et al., 2017; Yang et al., 2017; Tsilimigras et al., 2018). The changes in the gut microbiota may also differ within various gut niches, such as the mucosa-associated microbiota and the luminal microbiota (Galley et al., 2014a).

The time frame for the effects of stress on the gut microbiota varies, and recovery from stress-induced changes can vary, sometimes becoming persistent. For example, differences in microbial beta diversity were observed in mice after only 2 h of social stress, but a decrease in absolute abundance and relative abundance of *Lactobacillus* spp. was observed after 6 days (Galley et al., 2014b). The gut microbiome of infant rhesus monkeys altered significantly 3 days after being separated from their mothers and placed in individual cages, but returned to its pre-separation composition after 30 days (Zijlmans et al., 2015). In contrast, the stress-induced alterations in the gut microbiota of a GF mouse model did not fully reverse 21 days after the stress event, possibly due to other influences on gut microbiota besides the initial stress event (O'Mahony et al., 2009).

Several mechanisms have been suggested to mediate the interactions between the gut microbiota, stress, and mood. Alterations in the gut microbiota may affect behavior by inducing changes in the gut barrier, leading to increased gut permeability, and subsequently, the influx of gut-derived products into the bloodstream. This concept, known as “leaky gut,” suggests that increased gut permeability can lead to immune system activation and a heightened inflammatory state. This, in turn, may affect the central nervous system and contribute to mood alterations.

Indeed, several lines of evidence suggest that gut permeability can be altered by the gut microbiota. In a murine model of depression, it was observed that chronic stress led to an increase in gut permeability and that this effect could be mitigated by the administration of *Lactobacillus helveticus* and *Bifidobacterium longum* (Mayer, 2000). In another rodent model, the introduction of a specific commensal microbiota into GF mice, derived from conventionally raised mice, reduced stress-induced intestinal hyperpermeability and anxiety-like behavior (Castagliuolo et al., 1996). In humans, studies have linked increased gut permeability, as assessed by urinary excretion of orally administered lactulose and mannitol, to depression, depression scores, and severity of depressive symptoms, although this association is not always observed (Spitz et al., 1994; Santos et al., 2001; Lyte et al., 2011; Vogelzangs et al., 2013). Furthermore, in a study on individuals with irritable bowel syndrome, reduced tight junction gene expression was associated with higher levels of anxiety and depression (O'Brien et al., 2007).

Gut-derived products, such as lipopolysaccharides (LPS), may also impact mood by promoting an inflammatory state in the host. LPS is a component of the outer membrane of gram-negative bacteria and can trigger an immune response when it crosses the gut barrier and enters the bloodstream. Circulating LPS activates the immune system, leading to the release of proinflammatory cytokines. In rodents, both acute and chronic stressors have been associated with increased levels of circulating LPS, while stress during pregnancy results in increased LPS levels in dams and offspring (Mikocka-Walus et al., 2016; Jašarević et al., 2017). Stress-induced increases in LPS are paralleled by higher levels of proinflammatory cytokines, such as interleukin-6 (IL-6) and tumor necrosis factor-alpha (TNF-α), which have also been implicated in mood disorders (Bajaj et al., 2012; Shen et al., 2017; Fattorusso et al., 2019).

Increased LPS levels have been associated with behavioral alterations. LPS administration in rodents has been shown to induce sickness behavior, a cluster of behavioral changes that overlap with depressive-like behaviors, including reduced exploration, increased immobility in the forced swim test, and reduced sucrose preference (Gaykema et al., 1998; Ait-Belgnaoui et al., 2012; Erny et al., 2015; Rajkumar et al., 2015). In a human study, higher levels of LPS were related to more severe depressive symptoms, and serum levels of LPS-binding protein (LBP), which binds to LPS and facilitates its detection by the host’s immune system, have been associated with elevated depression scores (Kittana et al., 2018). In addition, the introduction of a microbiota from conventionally raised mice into GF mice resulted in decreased LPS levels in plasma and reduced anxiety-like behavior (Castagliuolo et al., 1996).

Immune signaling pathways represent a potential mediator between the gut microbiota, stress, and mood. Activation of the immune system and elevated proinflammatory cytokine levels have been related to mood disorders. Both peripheral cytokines and central cytokines, which are produced in the brain, can influence the brain and behavior. Peripheral cytokines can access the brain through various mechanisms, including active transport and diffusion through areas with a leaky blood–brain barrier or areas lacking an effective blood–brain barrier. Elevated proinflammatory cytokines have been associated with mood disorders and sickness behavior (Qiu et al., 1999; Maes et al., 2008; Smith and Garrett, 2011; Bharwani et al., 2017). Interestingly, the activation of microglia, the resident immune cells in the brain, has been implicated in the induction of depressive-like behaviors in rodents (Maes et al., 2013).

In summary, stress can induce changes in the gut microbiota composition, which can influence behavior through multiple potential mechanisms, such as altering gut permeability and promoting an inflammatory state.

## 4 Microbiota in leptospirosis: implications for susceptibility and pathogenesis

### 4.1 The microbiota’s role in leptospirosis

**Figure 2:**
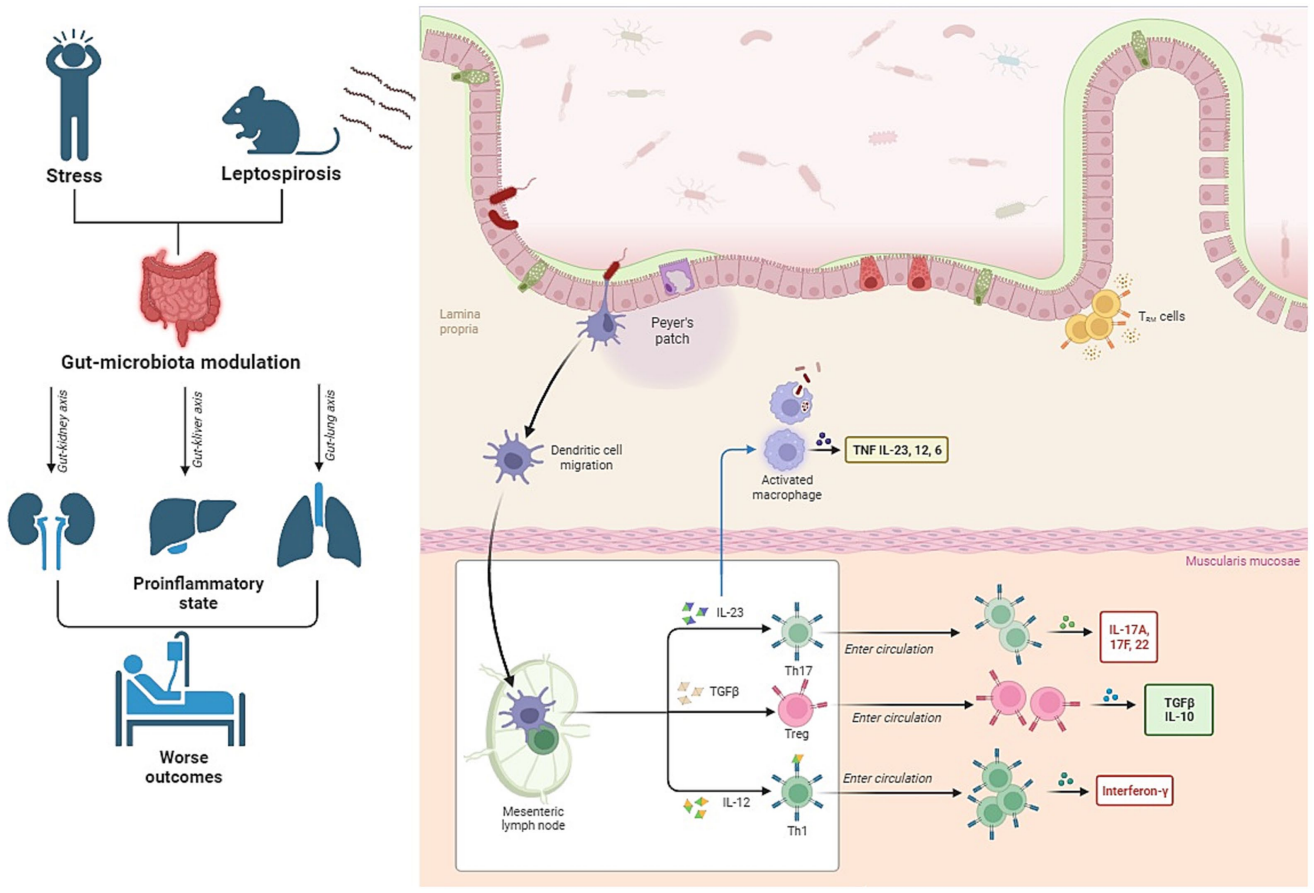
Interconnection between gut microbiota, stress, and leptospirosis. The figure illustrates that patients experiencing stress exhibit alterations in their gut microbiota. Additionally, recent animal studies have shown that leptospirosis, a bacterial infection, also leads to changes in the microbiota. The combined impact of these two conditions can potentially exacerbate inflammation during leptospirosis and result in damage to vital organs such as the kidneys, lungs, and liver through the so-called “axes”.

In the context of leptospirosis, a bacterial infection triggered by *L. interrogans*, the gut microbiota plays a crucial role (Figure 2). A balanced and healthy gut environment, shaped by a robust and well-functioning microbial ecosystem, is pivotal for guiding the immune system toward equilibrium (Rooks and Garrett, 2016; Burrello et al., 2018). Previous research has increasingly connected the gut microbiota to the heterogeneous susceptibility of hosts to various diseases (Zhang et al., 2020). A study by Alavi et al. provided strong evidence that interpersonal variations in the gut microbiome can determine an individual’s susceptibility or resistance to cholera infection (Alavi et al., 2020). The composition of the gut microbiota also appears to be a factor in explaining clinical symptoms in leptospirosis patients.

Some infections caused by nonenteropathogens, such as *Mycobacterium tuberculosis*, *Influenza*, and *Burkholderia pseudomallei*, have been shown to impact the microbiota’s composition (Winglee et al., 2014; Lankelma et al., 2017; Zhang et al., 2020). A study by Jacobson et al. demonstrated that it’s the community composition of the microbiota, rather than species richness, that plays a pivotal role in microbiota-mediated colonization resistance against pathogenic infections (Jacobson et al., 2018).

In a groundbreaking study conducted by Xufeng Xie and their team, the intricate interplay between the gut microbiota and leptospirosis was investigated (Xie et al., 2022). This study marked the first exploration into the role and underlying mechanisms of the gut microbiota in the context of leptospirosis. Notably, when the gut microbiota was depleted in mice, it led to weight loss and an increased leptospiral load in various organs compared to control mice. However, the effects were reversed when fecal microbiota transplantation (FMT) was performed on the microbiota-depleted mice. Moreover, the microbiota-depleted mice exhibited diminished phagocytosis and inflammatory responses in certain types of macrophages following infection. These findings suggest that the intestinal microbiota has a crucial role in modulating the host’s immune response to leptospirosis. It affects the phagocytic capabilities and inflammatory responses of specific macrophages, and its disruption can result in an increased leptospiral burden and dissemination in the infected host.

Furthermore, as evidenced by another study, host immune activation can lead to rapid transcriptional and metabolic adaptations in intestinal microbes (Becattini et al., 2021). Infection-induced disturbances in the intestinal environment disrupt the balance between intestinal immunity and the microbiota, ultimately leading to shifts in the population sizes of specific bacterial species (Rooks and Garrett, 2016).

### 4.2 Impact of intestinal bacteria on T-lymphocytes: immunoregulatory bacteria

Within the context of understanding how changes in the gut microbiota can influence T-lymphocytes and their impact on inflammation, our exploration leads us to delve deeper into the specific roles played by individual representatives of the intestinal microbiota (Shim et al., 2023).

Numerous studies have unraveled the intricate crosstalk between the gut microbiota and immune cells, particularly in relation to T-lymphocytes. The commensal microbiota, which comprises the community of microorganisms inhabiting the gut, has been found to exert a profound influence on the function, development, and differentiation of T cells, thus contributing to the maintenance of immune homeostasis.

CD4+ T cells, a subtype of T-lymphocytes, differentiate into various Th (T helper) lineages, each with distinct effector functions. Researchers have shown that certain members of the *Klebsiella* genera, such as *K. aeromobilis* and *K. pneumoniae*, are capable of inducing Th1 cell responses within the gut environment (Shim et al., 2023). Colonization of the gut by these Klebsiella species has been found to enhance the proliferation of Th1 cells, thereby augmenting their presence in the intestine. Additionally, probiotic bacteria, such as certain Lactobacillus strains, have demonstrated the ability to modulate Th1 cell activity (Takeda et al., 2011; Matsusaki et al., 2016). For example, *L. plantarum* and *L. sali*var*ius* were found to enhance the production of Th1 cytokines, such as tumor necrosis factor alpha (TNFα) and interferon gamma (IFNγ; Won et al., 2011). This influence of probiotic bacteria on Th1 cell activity signifies the potential for the gut microbiota to impact both Th1 and Th2 cell functions.

Moreover, the influence of the microbiota extends to Th2 cells, which secrete cytokines like IL-4, IL-5, and IL-13, playing a significant role in humoral immunity and defense against helminth infections, while also contributing to chronic inflammatory diseases (Walker and McKenzie, 2018). Studies have revealed that Lactobacillus strains and *B. fragilis* can inhibit Th2 activity by positively influencing Th1 cell responses, thereby modulating the balance between these two subsets of T cells (Mazmanian et al., 2005; Won et al., 2011).

Another critical subtype of T-lymphocytes is the Th17 cells, known for producing the proinflammatory cytokine IL-17 and playing a role in the pathogenesis of inflammatory and autoimmune diseases (Won et al., 2011). Th17 cells are typically absent in GF mice but become inducible upon microbial colonization. Specific bacteria, such as segmented filamentous bacteria (SFB) and various gram-positive species, have been identified as inducers of Th17 cell differentiation (Atarashi et al., 2008, 2015; Lee and Kim, 2017). These bacteria activate specific cell subsets in the lamina propria, promoting the differentiation of Th17 cells (Atarashi et al., 2015; Schnupf et al., 2015). Additionally, microbial bile acid metabolites can affect Th17 cell differentiation, highlighting the role of the microbiota in modulating these important T-lymphocytes (Hang et al., 2019).

Regulatory T cells (Treg cells) are integral for preventing autoimmune diseases and maintaining immune homeostasis (Dominguez-Villar and Hafler, 2018). Certain microbes, such as *B. fragilis*, *Bifidobacterium* strains, and various Lactobacillus strains, are known to influence Treg cell populations, leading to the development of Foxp3+ Treg cells that produce immune-regulatory cytokines like IL-10 (Round and Mazmanian, 2010). Dysbiosis due to factors like antibiotic treatment or changes in microbial composition can affect Treg cell generation and activity, further emphasizing the crucial relationship between the gut microbiota and autoimmune diseases (O'Mahony et al., 2008).

The realm of CD8+ T cells, essential for immune defense against intracellular pathogens and tumor surveillance, also experiences the impact of the gut microbiota (Zhang and Bevan, 2011). Specific probiotic species have been found to determine the anti-tumor efficacy of CD8+ T cells. Additionally, microbial byproducts, such as short-chain fatty acids (SCFAs) like butyrate and propionate, have been shown to influence CD8+ T cell activity by either inhibiting or promoting their activation, depending on the context (Nastasi et al., 2017).

### 4.3 Interactions along the gut-kidney, and gut-liver axes

Weil’s syndrome, a severe variant of leptospirosis accounting for approximately 10% of cases, is characterized by significant hepatic dysfunction coupled with renal failure and hemorrhages. Currently, there is a growing understanding of the intricate interplay between the gut microbiome and the health of the kidneys and liver, resulting in two vital axes known as the gut-kidney and gut-liver axes.

When the microbial communities within the gut fall out of balance, it can lead to a condition called intestinal dysbiosis, often as a result of breaches in the intestinal barrier. Additionally, viable bacteria may traverse from the gut into other sites beyond the intestines, including the kidneys. This phenomenon of bacterial translocation is often associated with issues such as bacterial dysbiosis, bacterial overgrowth, and weakened host immune defenses (Berg, 1999; Mielcarek et al., 2011).

Within the context of chronic kidney disease (CKD), it is noteworthy that the gut microbiota produces numerous uremic solutes and toxins, such as indoxyl sulfate, p-cresyl sulfate (PCS), and trimethylamine (TMA) N-oxide. Paradoxically, elevated urea concentration can lead to changes in the intestinal microbiota composition (Hobby et al., 2019). These uremic toxins can contribute to various health complications in CKD patients, encompassing renal anemia, pruritus, fatigue, mineral bone disorders, neurological impairments, and cardiovascular issues (Hobby et al., 2019).

The dynamic interaction between the gut microbiota and kidney diseases, known as the gut-kidney axis, is implicated in a wide spectrum of clinical manifestations, including CKD, acute kidney injury (AKI), hypertension, nephrolithiasis, immunoglobulin A (IgA) nephropathy, hemodialysis, and peritoneal dialysis (Al Khodor and Shatat, 2017; Hobby et al., 2019).

In recent years, a substantial focus has been directed toward understanding the intricate relationship between the gut microbiota and the liver, a two-way connection referred to as the gut-liver axis. This connection is facilitated through the portal vein and the biliary tract, enabling gut-derived metabolites to reach the liver. Simultaneously, the liver releases bile acids and other mediators back into the intestine (Di Tommaso et al., 2021). The integrity of the intestinal barrier, composed of various structural components such as the mucus layer, epithelial cells, vascular barrier, immune cells, and soluble mediators, plays a pivotal role in regulating this interaction, effectively limiting the systemic spread of toxins and pathogenic molecules (Albillos et al., 2020). While bacterial translocation, defined as the movement of bacteria and their products across the intestinal barrier into mesenteric lymph nodes (MLNs) or the portal venous system, is a physiological process crucial for immune system development, in normal conditions, only small amounts of bacteria and their products escape surveillance by resident immune cells, such as Kupffer cells, dendritic cells, natural killer (NK) cells, and lymphocytes (Garcovich et al., 2012; Nicoletti et al., 2019).

Consequently, any disturbances in the gut microbiota and alterations in the intestinal barrier are closely linked to the development and progression of liver diseases. Multiple studies have demonstrated a significant reduction in gut microbial diversity in individuals with liver disorders, along with an increased presence of pathogenic taxa like *Fusobacteria*, *Proteobacteria*, *Enterococcaceae*, and *Streptococacceae*, coupled with a depletion of beneficial microorganisms like *Bacteroidetes*, *Ruminococcus*, *Roseburia*, *Veillonellaceae*, and *Lachnospiraceae* (Gómez-Hurtado et al., 2016). Notably, cirrhotic patients exhibit an inverse correlation between the beneficial bacteria-to-pathogenic bacteria ratio, known as the cirrhosis/dysbiosis ratio (CDR), and the model for end-stage liver disease (MELD) score and endotoxin levels (Bajaj et al., 2014).

The reduction of beneficial autologous taxa leads to decreased production of short-chain fatty acids (SCFAs), resulting in the conversion of primary bile acids into secondary bile acids, which further exacerbates gut dysbiosis, weakens the integrity of the intestinal barrier, reduces gut motility, and promotes small intestinal bacterial overgrowth (SIBO; Rocco et al., 2021). These changes amplify the rate of bacterial translocation and promote endotoxemia, introducing a significant amount of pathogen-associated molecular patterns (PAMPs) into the MLNs. This, in turn, leads to their spread to the liver via the portal circulation (Cirera et al., 2001; Muñoz et al., 2012).

Upon reaching the liver, PAMPs interact with resident immune cells like Kupffer cells via TLRs, triggering MyD88-dependent and MyD88-independent molecular pathways that activate NF-kB. This results in the release of inflammatory cytokines such as TNF-α, IL-1β, IL-6, IL-18, as well as chemokines, NO, and reactive oxygen species (Seki and Schnabl, 2012).

## 5 Biogenic amines during stress: effects on lymphocytes and inflammation

### 5.1 The influence of biogenic amines on lymphocyte function

#### 5.1.1 Adrenergic receptors

**Figure 3:**
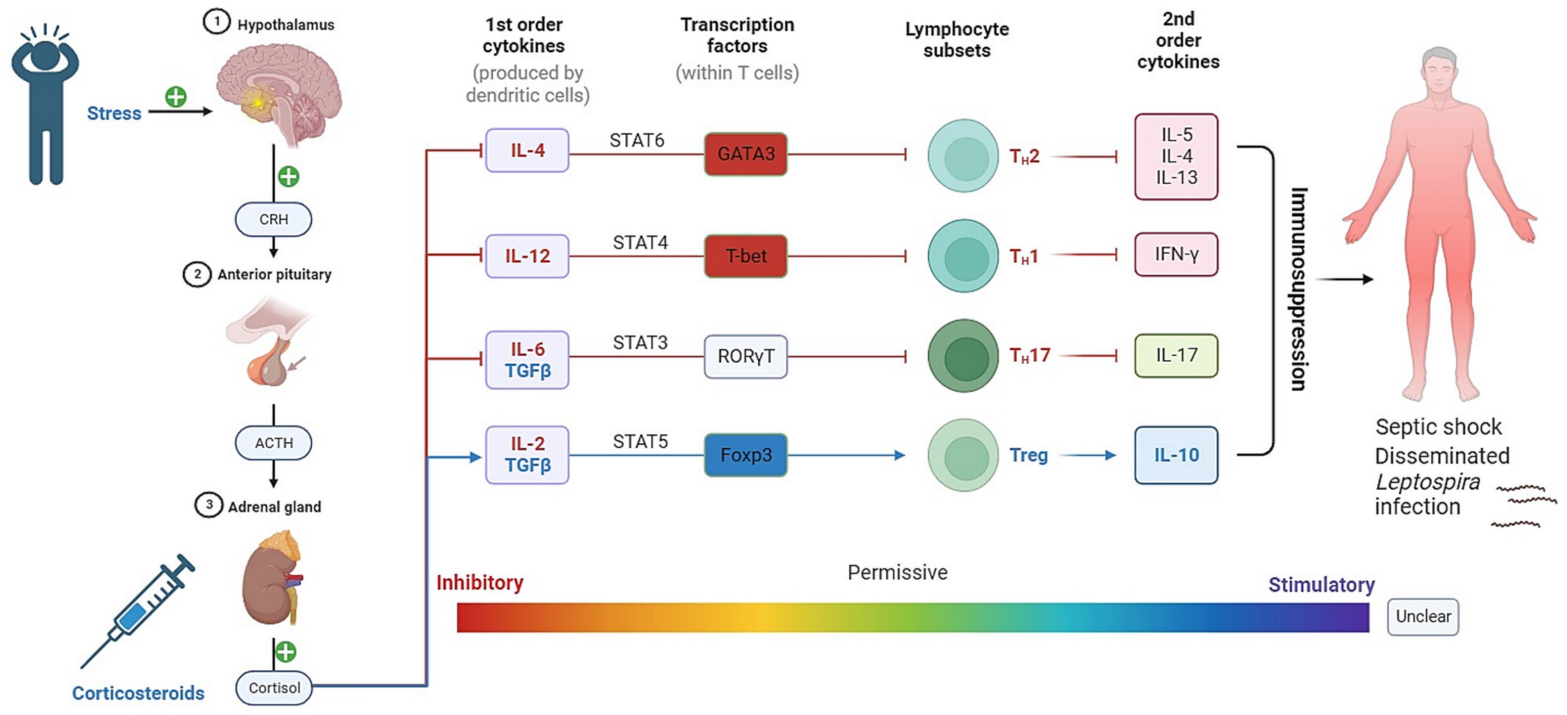
Impact of GCs on T-helper cell differentiation and its implications for leptospirosis. In addition to their role in stress response, GCs and catecholamines have direct effects on T lymphocytes and other immune cells via their respective receptors. These can suggest that the interplay between stress hormones and immune cells plays a crucial role in shaping the immune response during leptospirosis infection.

The catecholamines, including norepinephrine (NE) and epinephrine, have significant roles to play in their interactions with T-lymphocytes in the human body (Figure 3). In the plasma, both NE and dopamine are present, with NE constituting approximately 20% of the quantity of epinephrine and dopamine (Van Loon, 1983). Notably, T-lymphocytes account for about 70% of peripheral blood mononuclear cells (PBMCs) in the intravascular space (Autissier et al., 2010).

In the spleen, sympathetic innervation is regional and specific, primarily found in the white pulp surrounding central arteries, which predominantly comprises T-lymphocytes (Felten et al., 1985; Kraal, 1992). Additionally, catecholamines may interact with T-lymphocytes in the central nervous system, especially when inflammation compromises the blood–brain barrier, allowing T-lymphocytes to enter and potentially interact with catecholamines (Varatharaj and Galea, 2017).

Understanding the functional impact of catecholamines on T-lymphocytes necessitates a comprehensive exploration of adrenergic receptor (AR) expression. Examining the literature reveals a complex landscape of AR subtypes expressed on T-lymphocytes.

##### 5.1.1.1 α-adrenergic receptors

Early studies initially indicated that T-lymphocytes did not express α-ARs, and there was a reported absence of α1-AR mRNA in PBMCs (Casale and Kaliner, 1984; Cook-Mills et al., 1995). However, recent research has shown the presence of α1-AR mRNA in PBMCs (Tayebati et al., 2000). Experiments demonstrated that α1-AR agonists like phenylephrine reduced H3-thymidine incorporation in T-lymphocytes in a dose-dependent manner, which was reversed by α-AR antagonists (Heilig et al., 1993). The expression of α1-ARs on T-lymphocytes seems to occur mainly upon activation with mitogens such as phytohemagglutinin (PHA) or lipopolysaccharide (LPS) [83]. In these activated T-lymphocytes, exposure to norepinephrine resulted in increased ERK activation, which could be mitigated by selective α1-AR antagonism (Rouppe van der Voort et al., 2000). Other studies have demonstrated that phenylephrine treatment of PBMCs from patients with juvenile rheumatoid arthritis led to cytokine alterations, including increased IL-6 production compared to healthy control PBMCs (Heijnen et al., 1996). The proliferation and cytokine production of pan T-lymphocytes were not affected by non-specific activation with the mitogen concanavalin A (ConA) followed by phenylephrine treatment (Bao et al., 2007). However, with ConA-activated pan T-lymphocytes, an intriguing study showed that treatment with an MAO inhibitor led to a shift toward Th2 polarization with more IL-4 production, a phenomenon blocked by α1-AR and β2-AR antagonists but not α2-AR or β1-AR antagonists (Huang et al., 2015). Additionally, the intracellular redox environment was observed to be modulated by α1-AR agonism and antagonism, providing further evidence of the presence of α1-ARs on T-lymphocytes (Case et al., 2016). As discussed earlier, α2-ARs function through a distinct intracellular cascade, with clonidine-induced agonism in ConA-activated pan T-lymphocytes inhibiting proliferation and reducing IFN-γ and IL-4 production. This inhibition could be mitigated by α2-AR antagonism and partly attenuated by the inhibition of PLC or PKC, highlighting the role of this pathway in α2-induced T-lymphocyte inhibition (Bao et al., 2007). The CD4+ T-lymphocyte redox environment was also found to be affected by α2-ARs, adding to the complexity of AR signaling in these adaptive immune cells (Case et al., 2016).

In summary, the literature reveals a complex picture of α-AR expression on T-lymphocytes, with contradicting findings across different studies. These receptors appear to play a role in modulating T-lymphocyte function, influencing their cytokine production, proliferation, and redox status. However, the *in vivo* relevance of these mechanisms, particularly in patients taking systemic α-AR modulating drugs, warrants further investigation.

##### 5.1.1.2 β-adrenergic receptors

β-AR expression was initially indicated indirectly through the increased cAMP levels upon application of catecholamines, suggesting the presence of β-ARs on T-lymphocytes. Subsequently, different subtypes of β-ARs were explored individually (Bourne and Melmon, 1971; Makman, 1971; Bach, 1975).

Evidence of β1-AR expression on T-lymphocytes is limited, but certain reports suggest a functional role for this receptor in effector T-lymphocytes. One study demonstrated that NE suppressed IFN-γ and TNF-α production in murine intestinal intraepithelial CD3+ T-lymphocytes through β1-AR activation, which was confirmed by selective pharmacological activation and blockade (Takayanagi et al., 2012). β1-AR expression on Tregs has also been discussed (Cosentino et al., 2007). Moreover, reports indicate a greater expression of β1-ARs on Tregs compared to CD25− T-lymphocytes in healthy human patients exposed to acute physical stressors (Freier et al., 2010). β3-AR mRNA has been detected in ConA-stimulated pan T-lymphocytes, but agonism at β3-AR did not result in any functional changes, potentially because β3-AR agonism generates less cAMP than other β-AR subtypes (Knapp et al., 1997).

In contrast, β2-AR expression on T-lymphocytes has been widely acknowledged. Naïve CD4+ T-lymphocytes were found to express “high-affinity, saturable β2-ARs,” with this expression tightly regulated as T-lymphocytes differentiate (Kohm and Sanders, 2001). CD8+ T-lymphocytes express β2-ARs in greater quantities than CD4+ cells, and these expressions are differentially regulated in both healthy and rheumatoid arthritis (RA) patients (Baerwald et al., 1997; Wahle et al., 2001). When T-lymphocytes polarize to TH1 and TH2 lineages, the expression levels of β2-AR are increased or decreased, respectively (McAlees et al., 2011). Exposure of naïve CD4+ T-lymphocytes to NE or selective β2-AR agonists led to reduced IL-2 and IFN-γ production upon subsequent activation (Kin and Sanders, 2006). Interestingly, the cytokine response varied with the timing of NE addition, with NE preceding TH1 polarization leading to increased IFN-γ production from these cells (Kin and Sanders, 2006). The intricate regulation of NE may allow it to play both suppressive and activating roles in the T-lymphocyte inflammatory response, potentially attributed to the intracellular role of cAMP during different activation states or non-canonical β-AR mechanisms (Case and Zimmerman, 2016).

#### 5.1.2 Dopamine receptors

Dopamine receptors (DRs) of all subtypes, including D1-D5, have been identified on various T-lymphocyte subtypes, with dynamic and context-dependent expression levels.

D1-like receptor agonism with physiological concentrations of dopamine *in vitro* has been shown to impair the cytotoxicity and reduce the proliferation of CD4+ and CD8+ cells, as well as suppress their proliferation upon IL-2 induction (Saha et al., 2001). D1-like family activation has also been associated with the polarization of naïve CD4+ T-lymphocytes to TH2 in response to dopamine delivered by dendritic cells (DCs) in a dose-dependent manner (Nakano et al., 2009). Other studies have highlighted the role of D1-like receptors in TH17 polarization mediated by IL-23 production by DCs (Nakano et al., 2008). D1-like receptor activation in Tregs resulted in reduced production of IL-10 and TGF-β, as well as decreased Treg proliferation (Cosentino et al., 2007). These cytokines are vital for Tregs to suppress effector T-lymphocyte proliferation, and dopamine appears to inhibit these processes. High concentrations of dopamine can also effectively inhibit the proliferation and IFN-γ synthesis in activated effector T-lymphocytes (Bergquist et al., 1994; Cosentino et al., 2007).

In summary, D1-like receptor activation appears to decrease the functionality of CD4+ T-lymphocytes and inhibit the ability of Tregs to suppress effector T-lymphocytes.

D2-like family receptors display less consistent behavior across receptor subtypes. Activation of D2R and D3R on CD8+ T-lymphocytes has been linked to increased adhesion to fibronectin and the promotion of cellular trafficking and adhesion (Levite et al., 2001). Dopamine agonism at D3R in naïve CD8+ T-lymphocytes has been associated with increased adhesion to fibronectin and Intercellular Adhesion Molecule 1 (ICAM-1/CD54), as well as increased T-lymphocyte proliferation (Watanabe et al., 2006). D2R and D3R activation has led to increased expression of IL-10 and TNF-α, respectively (Besser et al., 2005). The expression of IL-10 inhibits effector T-lymphocytes, while D3R-mediated chemotaxis promotes increased CD8+ cell function, creating a complex and contradictory outcome. D4R activation, on the other hand, results in quiescence by inhibiting ERK1/ERK2 phosphorylation, upregulating Kruppel-like factor 2 (KLF-2), and mimicking the suppressive effects of the D1-like family (Sarkar et al., 2006).

In conclusion, dopamine’s role in T-lymphocyte physiology is indeed multifaceted, with the potential to enhance the homing and chemotaxis of CD8+ cells, alter CD4+ cell polarization, inhibit activated effector T-lymphocytes, and suppress Treg cells. These effects vary based on the dopamine receptor subtypes and their affinities for dopamine, creating a complex and dynamic interplay. It is also essential to recognize that T-lymphocytes interact directly with other cell types, such as dendritic cells, which also utilize dopamine in their regulation (Pacheco et al., 2009).

## 6 Biogenic amines’ influence on microbiota and Leptospira: quorum sensing

Quorum sensing (QS) serves as a pivotal regulatory mechanism, orchestrating various bacterial activities, including sporulation, biofilm production, secretion of virulence factors, and diverse interactions among bacteria. These interactions encompass interspecies competition, cooperative actions, and even the recognition of kinship among bacteria.

Within the intricate web of the gut ecosystem, molecules like epinephrine and norepinephrine, responsible for functions such as regulating gut motility, controlling potassium and chloride secretion, enhancing epithelial barrier function, and influencing inflammatory responses, play an unexpectedly intriguing role (Eisenhofer et al., 1997; Asano et al., 2012). These molecules are not just confined to their traditional physiological roles but are also recognized by bacterial QS receptors. Their interaction with these receptors leads to profound alterations in bacterial behavior.

Epinephrine and norepinephrine have been observed to share a signaling pathway with bacteria that employ QS signal AI-3 through the action of two-component systems like QseC/B and QseE/F (Sperandio et al., 2003; Clarke et al., 2006; Hughes et al., 2009). These hormones are recognized as host-derived AI-3 mimics, and their presence significantly impacts the signal reception of AI-3. This results in the activation of virulence-related processes in bacteria such as *Escherichia coli* O157:H7 and *Salmonella* (Clarke et al., 2006; Kim et al., 2020).

Notably, another stress hormone found in mice intestinal mucosa, dynorphin, has been identified to activate QS signaling in *Pseudomonas aeruginosa* (Zaborina et al., 2007). This activation leads to an enhancement of the bacterium’s virulence within the host environment.

In an article by Karukriti Kaushik Ghosh et al., they examined the impact of the host stress hormone catecholamine on Leptospira gene transcripts encoding outer membrane proteins (Ghosh et al., 2018). While catecholamine supplementation did not affect the *in vitro* growth of *Leptospira interrogans*, it resulted in differential transcription of 7 out of 41 genes, which could be reversed by the antagonist propranolol. They also studied LIC20035/LB047, a differentially regulated protein, which was found to be immunogenic and capable of adhering to host extracellular matrices. This protein was surface-exposed on the outer membrane, and the recombinant LIC20035 could be serologically detected in leptospirosis-positive human and bovine sera. Additionally, it showed a strong affinity for binding to various host extracellular matrices, particularly collagen and chondroitin sulfate.

## 7 T helper cell differentiation and GCs

**Figure 4:**
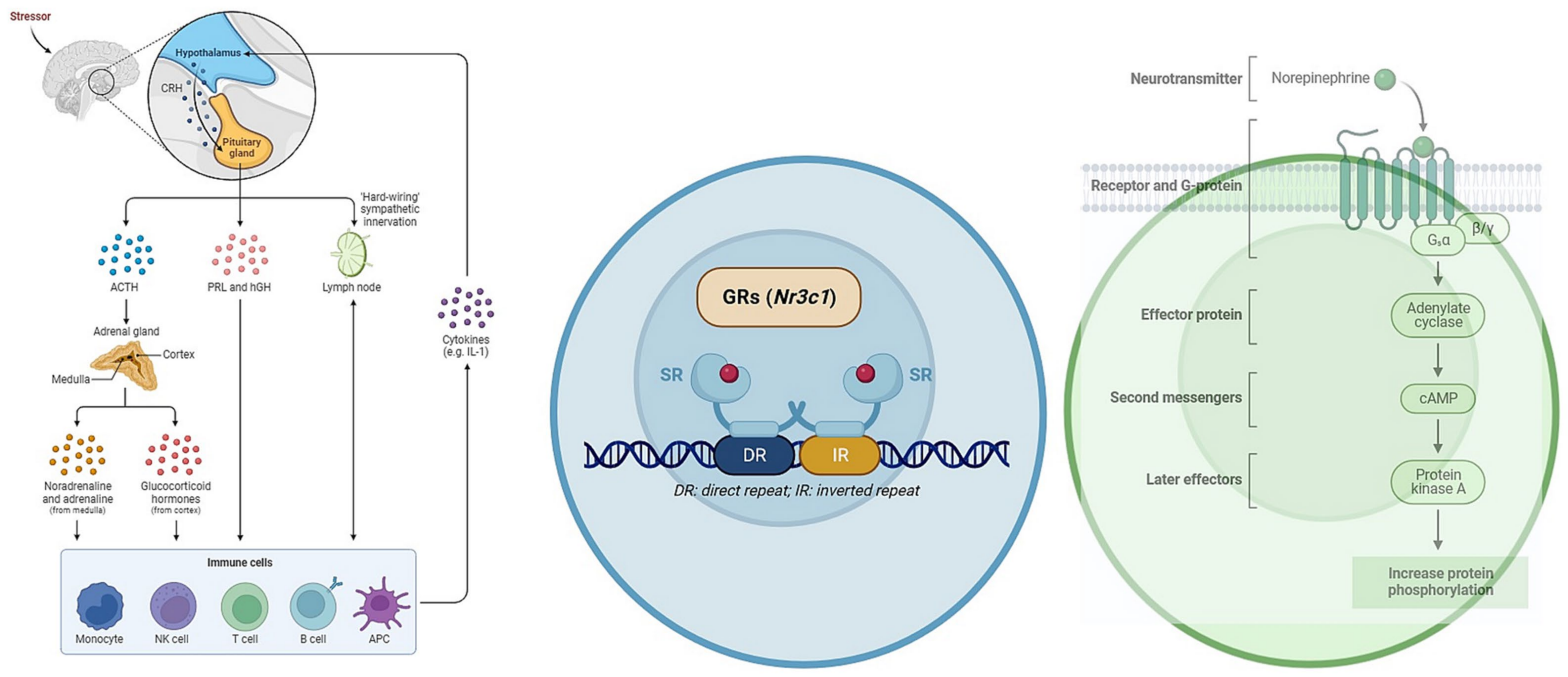
The role of GCs and catecholamines in immunoregulation. The figure illustrates the impact of glucocorticoids (GCs) and catecholamines on immunoregulation. It demonstrates how cortisol, released through the hypothalamic–pituitary–adrenal (HPA) axis, influences the release of cytokines and transcription factors in T-lymphocytes. This immunosuppressive effect can contribute to the spread of Leptospira and exacerbate the severity of the disease.

Glucocorticoids (GCs) represent integral components of the physiological stress response and play a pivotal role in immune system modulation (Figures 3, 4). This chapter delves into the profound influence of GCs on T helper cells, a heterogeneous class of immune cells responsible for orchestrating diverse immune responses.

The glucocorticoid receptor (GR) is expressed in all T cells, but the extent of GC sensitivity varies significantly among different cell populations. Consequently, GCs exert selective suppression of specific T helper cell responses over others. They effectively suppress inflammatory T helper 1 (Th1) cell responses while moderately inhibiting Th2 cell responses and, intriguingly, permitting the development of IL-17-producing T helper (Th17) cells.

The initial polarization of T helper cells depends on signals originating from innate immune cells, and GCs impact T cell differentiation by regulating cytokine synthesis at this early stage. This regulation, in turn, directs T helper cell differentiation. For instance, GCs potently inhibit the production of interleukin-12 (IL-12) and interferon-gamma (IFNγ) by macrophages and dendritic cells, thereby reducing Th1 cell induction (Li et al., 2015; Oh et al., 2017). This occurs through the inhibition of STAT4 phosphorylation and STAT1 gene expression, thus preventing Th1 cell differentiation (Franchimont et al., 2000; Hu et al., 2003). GCs furthermore inhibit expression of T-bet (Tbx21) and IFNγ (Ifng) genes, and the GR directly associates with T-bet protein to prevent expression of a Th1 cell transcriptional program (Liberman et al., 2007; de Castro et al., 2018).

In the case of Th2 cell differentiation, GCs exert suppressive effects, although to a lesser extent than on Th1 cells. Inhibition of IL-12 and IFNγ production by innate cells, as a result of the prevention of Th1 cell differentiation, allows Th2 cells to proceed unhindered. While GCs have minimal impact on IL-4-induced STAT6 phosphorylation, they induce mitogen-activated protein kinase phosphatase 1 (MKP1), leading to the inhibition of p38 activation and GATA3 expression, ultimately preventing the expression of IL-4, IL-5, and IL-13 (Liberman et al., 2009; Maneechotesuwan et al., 2009).

In contrast, GCs promote the differentiation of Th17 cells. They synergize with IL-6-activated STAT3 to facilitate Th17 cell development (Zhang et al., 1997). These cells tend to be refractory to GCs, partly due to increased expression of the GC-exporting membrane channel MDR1 (Ramesh et al., 2014). GCs also enhance the expression of RORγt and IL-17 in Th17 cells, although they can suppress IL-22 and GM-CSF (Banuelos et al., 2016; de Castro et al., 2018). This hierarchy of GC effects results in a preference for Th17 cell responses.

Less is known about the regulation of Th9 and Th22 cells by GCs, but *in vitro* studies suggest that GCs suppress the secretion of their signature cytokines, IL-9 and IL-22, respectively (Holz et al., 2005). This suppression might be linked to the GR’s ability to inhibit PU.1 activity and aryl hydrocarbon receptor (AHR) expression, although further investigation is needed (Wang et al., 2009; Uhlenhaut et al., 2013).

T follicular helper cells’ response to GCs remains less clear, as GCs inhibit IL-21 production but upregulate BCL-6 expression in non-T cells (Linhares et al., 2013). Consequently, there is a discernible hierarchy of GC effects on T helper cell differentiation, with strong inhibition of Th1 cells, moderate inhibition of Th2 cells, and permissiveness for Th17 cell responses (Reddy et al., 2009). This hierarchy extends to T helper cell survival, with GCs causing more significant apoptosis in Th1 cells compared to Th2 and Th17 cells.

In contrast to T helper cells, extrathymic regulatory T (Treg) cell differentiation is significantly promoted by GC signaling (Galon et al., 2002). GCs enhance the upregulation of TGFβ receptors, FOXP3, and IL-10 (Karagiannidis et al., 2004). Moreover, the GR is upregulated during Treg cell differentiation, and GC-responsive genes induced by leucine zipper (Gilz) promote Treg cell differentiation (Bereshchenko et al., 2014; Schmidt et al., 2018). Transgenic overexpression of the GR in T cells has little impact on Treg cell numbers but significantly reduces T helper cell populations (Rocamora-Reverte et al., 2019). Furthermore, Treg cell-specific loss of GR exacerbates colitis (Rocamora-Reverte et al., 2019). These observations suggest that Treg cell differentiation and function are influenced by GCs, and that endogenous GCs may primarily effect immunosuppression by enhancing Treg cell activity during effector T cell responses. Additionally, Treg cells display greater resistance to GC-induced apoptosis, further emphasizing the pivotal role of GCs in supporting Treg cell differentiation and function (Tischner et al., 2012; Prenek et al., 2020).

## 8 GCs, TLRs, and Leptospira infection

The innate immune system stands as the host’s foremost line of defense, playing a crucial role in the initial recognition and elimination of invading leptospires (Fraga et al., 2011). Central to this recognition process are the Pattern Recognition Receptors (PRRs), which are expressed on the surface of innate immune cells like macrophages and dendritic cells (DCs). These receptors, including the Toll-like receptors (TLRs) and nucleotide-binding oligomerization domain (NOD)-like receptors (NLRs), serve to identify Microbial Pathogen-Associated Molecular Patterns (PAMPs; Akira et al., 2006). In the context of leptospirosis, considerable attention has been directed toward Toll-like receptors, particularly TLR2 and TLR4, within the TLR family. These receptors are key players in the innate immune system’s recognition and response to leptospires (Werts et al., 2001). In mice, which are resistant to leptospirosis, the LPS is recognized by both TLR2 and TLR4. Indeed, both TLR4 and TLR2 stimulation is important in controlling leptospirosis in mice. Infected with *L. interrogans*, double TLR2/TLR4 knockout mice died quickly from hepatic and renal failure (Nahori et al., 2005).

The influence of glucocorticoids (GCs) on the regulation of TLR2 expression remains a subject of discussion. Findings from various studies have reported conflicting outcomes, with some indicating downregulation and others suggesting upregulation of TLR2 (Hoppstädter et al., 2019; Ricci et al., 2021). Moreover, genetic variations in TLRs have been associated with variations in the severity of leptospirosis. Specific gene polymorphisms, such as TLR1 Ile602Ser and TLR2 Arg753Gln, have emerged as substantial factors affecting the development of severe leptospirosis, particularly cases characterized by jaundice and hepatic insufficiency (Cédola et al., 2015).

Corticosteroids, commonly employed for the treatment of inflammation in patients with leptospirosis, have demonstrated their effectiveness in improving the survival rates of these individuals (Trivedi et al., 2010; Schulze et al., 2014). However, it’s crucial to acknowledge that while these drugs can be beneficial in managing the disease, their potent immunosuppressive properties raise concerns. Excessive immunosuppression triggered by corticosteroids can potentially lead to severe consequences, including sepsis and the dissemination of leptospires throughout the body and may increase the risk of nosocomial infections (Duarte-Neto et al., 2019; Win et al., 2022). Therefore, the administration of corticosteroids in leptospirosis cases requires a careful and balanced approach to achieve the desired therapeutic effects while minimizing the risks associated with immunosuppression.

## 9 Conclusion

In conclusion, leptospirosis remains a significant public health concern with diverse implications for human populations. This review has shed light on the multifaceted relationship between stress and leptospirosis, elucidating potential mechanisms by which stress influences disease dynamics. Stress impacts leptospirosis through intricate pathways, including the modulation of the intestinal microbiome and the influence of immunoregulatory bacteria in T-lymphocyte modulation. Furthermore, this interaction is compounded by the direct effects of catecholamines and glucocorticoids on T lymphocytes, showcasing the intricate web of connections that underlie this interplay.

While our review has contributed valuable insights into the crosstalk between stress and leptospirosis, it is evident that this complex relationship remains far from fully understood. To comprehensively unravel the intricacies of how stress affects leptospirosis, it is imperative to embark on further investigations. Future research endeavors should delve into the nuanced interactions within the intestinal microbiome, explore the precise roles of immunoregulatory bacteria, and elucidate the direct mechanisms through which catecholamines and glucocorticoids influence T lymphocytes.

## Author contributions

PP: Conceptualization, Visualization, Writing – original draft. VO: Writing – review & editing. IK: Writing – review & editing. IB: Writing – review & editing. KL: Writing – review & editing. OK: Conceptualization, Visualization, Writing – original draft.
